# Central Retinal Artery Occlusion in Takayasu's Arteritis as the First Presentation of the Disease

**DOI:** 10.1155/2016/6492513

**Published:** 2016-10-27

**Authors:** Hande Guclu, Vuslat Pelitli Gurlu, Sadık Altan Ozal, Orkut Guclu

**Affiliations:** ^1^Ophthalmology Department, Trakya University of Medicine, Edirne, Turkey; ^2^Cardiovascular Surgery Department, Trakya University of Medicine, Edirne, Turkey

## Abstract

Takayasu's arteritis (TA) is a chronic inflammatory granulomatous vasculitis which affects large and medium arterial vessels. The disease involves especially subclavian arteries and aortic branches but it can consist of any arteries. The major pathology is granulomatous panarteritis with intima proliferation and defects of the elastic lamina of the vessels. We present a case of central retinal artery occlusion in TA as the first presentation of the disease. To the best of our knowledge, the present case is the first case that demonstrates central retinal artery occlusion as an initial manifestation in TA. A 48-year-old woman was admitted to our clinic with the complaint of sudden and painless vision loss in her right eye for one day. Although retinal artery involvement is a very rare presentation in TA, it is important to recall TA particularly in young patients with retinal artery occlusion.

## 1. Introduction

Takayasu's arteritis (TA) is a chronic inflammatory granulomatous vasculitis which influences large and medium arterial vessels. It is an unusual and idiopathic disease that affects aorta and its branches and causes constriction and obliteration [1–3]. The disease involves especially subclavian arteries and aortic branches but can affect any arteries. The main reason of the disease remains unknown. The major pathology is granulomatous panarteritis with intima proliferation and defects of the elastic lamina of the vessels [4].

The clinical presentation is related with the location of the involved artery. Patients with carotid artery involvement encounter decreased retinal perfusion leading to chronic ocular ischemia. Ocular ischemic syndrome and related ocular manifestations such as retinal microaneurysms, microhemorrhages, branch retinal vein occlusion, proliferative retinopathy, cataract, neovascular glaucoma, ischemic optic neuropathy, retinal detachment, and optic atrophy in TA were reported previously [1–3].

We present a case of central retinal artery occlusion in TA as the first presentation of the disease. To the best of our knowledge, the present case is the first case that demonstrates central retinal artery occlusion as an initial manifestation in TA.

## 2. Case Report

**Figure 1:**
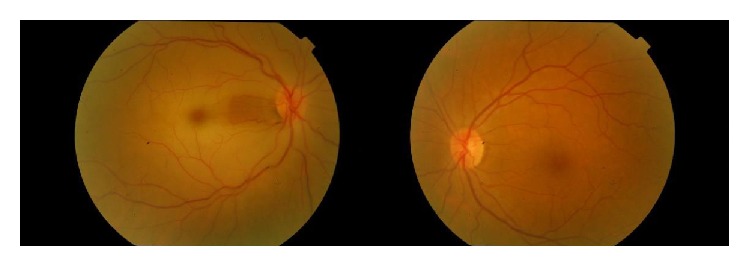
Initial photographs of the patient; central retinal artery occlusion in the right eye. Left eye is normal.

**Figure 2:**
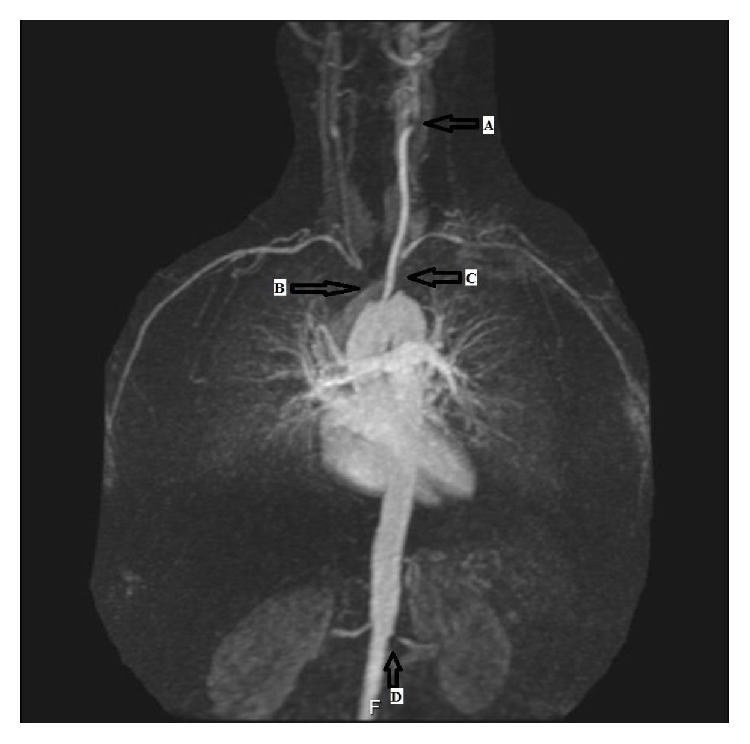
MR angiography of the patient (A: left carotid artery bifurcation stenosis, B: occlusion of brachiocephalic trunk, C: segmental stenosis of left subclavian artery, and D: left renal artery stenosis).

**Figure 3:**
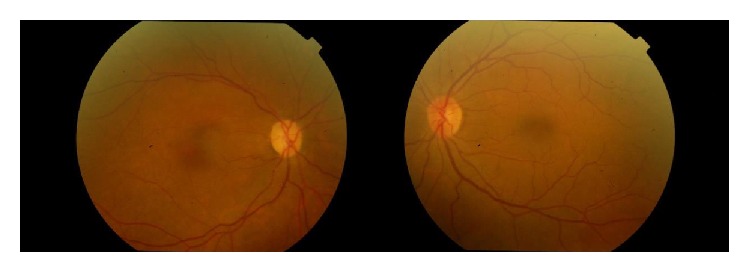
Fundus photographs of the patient after four months.

A 48-year-old woman was admitted to our clinic with the complaint of sudden and painless vision loss in her right eye for one day. She had no previous medical history and no history of neck pain, dizziness, or fever and drug usage. On ophthalmologic examination, there was just the light perception in the right eye and best corrected visual acuity (BCVA) was 20/20 in the left eye. Relative afferent pupillary defect (RAPD) was detected in the right eye. Anterior segment examination was normal bilaterally. Intraocular pressure was measured to be 16 mmHg and 17 mmHg in the right eye and in the left eye, respectively. Fundus examination in the right eye revealed pale retina with cherry red spot and left eye was normal. These findings supported the diagnosis of central retinal artery occlusion (Figure 1). Ocular massage was done; oral acetazolamide was administered to the patient. To establish the etiology of central retinal artery occlusion (CRAO), consultations were sought with the cardiovascular surgery, hematology, and rheumatology experts. In the systemic examination, the patient's upper extremity pulses were not palpable. Magnetic resonance (MR) angiography revealed segmental stenosis of the left carotid artery, brachiocephalic truncus, and the left subclavian occlusion (Figure 2). The patient was clinically diagnosed with TA and was referred to the rheumatology clinic. The patient received intravenous methylprednisolone 4 × 250 mg/kg per day for two days and continued with oral prednisone 48 mg per day and methotrexate 10 mg per day over four months. But there was no improvement in visual acuity of the right eye of the patient (Figure 3).

After 3 years, in ophthalmologic examination, there was no light perception in the right eye and BCVA was 20/20 in the left eye. Anterior segment examination was normal bilaterally. Intraocular pressure was measured to be 16 mmHg and 12 mmHg in the right eye and in the left eye, respectively. Fundus examination in the right eye revealed a pale optic disc.

## 3. Discussion

Takayasu's arteritis is an uncommon inflammatory disease of the large and medium sized arteries [2]. Ocular presentation of TA is reported to vary between 8.1% and 68% of the patients [4]. The ocular findings of the disease are related with carotid artery obliteration which leads to hypoperfusion of all the eye structures. Reduced blood flow in the carotid arteries causes Takayasu's retinopathy which is related to the chronic ischemia and occurs in the late phase of the disease [5].

Takayasu's arteritis is a disease that affects large vessels; however, previous reports presented the involvement of small retinal vessels. Occlusion of the retinal vessels is the other reason for ocular symptoms in TA. It is reported that the retinal vessel involvement is very rare presentation of the disease [4, 6]. Vasa vasorum supports the media and adventitia of the vessels. The initial inflammation around the vasa vasorum is the main histopathological feature in TA, which causes vascular inflammation and intimal proliferation [4]. Retinal vessel involvement, branch retinal artery occlusion, central retinal artery occlusion (CRAO), and branch retinal vein occlusion were demonstrated in previous reports [4, 6–10]. Kaushik et al. demonstrated branch retinal artery occlusion in a TA patient with left subclavian and common carotid artery occlusion [10].

Central retinal artery occlusion was the first clinical finding of the TA in our patient. The patient's upper extremity pulses were not palpable. Left carotid artery has segmental stenosis and brachiocephalic truncus and left subclavian occlusion was present in our case as the radiologic features of TA. To the best of our knowledge, the present report is the first case report that demonstrates central retinal artery occlusion in TA as the initial presentation of the disease.

Although retinal artery involvement is a very rare presentation in TA, it is important to recall TA particularly in young patients with retinal artery occlusion.
